# Mucosa-Associated Bacterial Diversity in Necrotizing Enterocolitis

**DOI:** 10.1371/journal.pone.0105046

**Published:** 2014-09-09

**Authors:** Rachel Brower-Sinning, Diana Zhong, Misty Good, Brian Firek, Robyn Baker, Chhinder P. Sodhi, David J. Hackam, Michael J. Morowitz

**Affiliations:** 1 Division of Pediatric Surgery, Children's Hospital of Pittsburgh, Pittsburgh, Pennsylvania, United States of America; 2 Division of Newborn Medicine, Children's Hospital of Pittsburgh, Pittsburgh, Pennsylvania, United States of America; 3 Department of Surgery, University of Pittsburgh School of Medicine, Pittsburgh, Pennsylvania, United States of America; 4 Department of Pediatrics, University of Pittsburgh School of Medicine, Pittsburgh, Pennsylvania, United States of America; Radboud University Medical Centre, NCMLS, Netherlands

## Abstract

**Background:**

Previous studies of infant fecal samples have failed to clarify the role of gut bacteria in the pathogenesis of NEC. We sought to characterize bacterial communities within intestinal tissue resected from infants with and without NEC.

**Methods:**

26 intestinal samples were resected from 19 infants, including 16 NEC samples and 10 non-NEC samples. Bacterial 16S rRNA gene sequences were amplified and sequenced. Analysis allowed for taxonomic identification, and quantitative PCR was used to quantify the bacterial load within samples.

**Results:**

NEC samples generally contained an increased total burden of bacteria. NEC and non-NEC sample sets were both marked by high inter-individual variability and an abundance of opportunistic pathogens. There was no statistically significant distinction between the composition of NEC and non-NEC microbial communities. K-means clustering enabled us to identify several stable clusters, including clusters of NEC and midgut volvulus samples enriched with *Clostridium* and *Bacteroides*. Another cluster containing both NEC and non-NEC samples was marked by an abundance of *Enterobacteriaceae* and decreased diversity among NEC samples.

**Conclusions:**

The results indicate that NEC is a disease without a uniform pattern of microbial colonization, but that NEC is associated with an abundance of strict anaerobes and a decrease in community diversity.

## Introduction

NEC remains a significant cause of morbidity and mortality among low birth weight infants [1]. Although intestinal microbes are believed to contribute to the pathogenesis of the disease, the details of this relationship remain poorly understood [2]. Many culture-based and culture-independent investigations have been conducted to contrast the microbiota of infants with and without NEC. Although some bacterial genera, e.g. *Klebsiella* and *Clostridium*, have commonly been associated with the disease, no individual causative pathogen and no single pattern of perinatal colonization has been specifically and reproducibly associated with NEC [2]. Fortunately, the recent advent of high-throughput molecular assays has created opportunities to gain insights into NEC pathogenesis by acquiring culture-independent profiles of intestinal microbes from progressively larger cohorts of infants.

Several studies have contributed to an understanding of the microbiota of premature infants by profiling 16S rRNA gene sequences present within fecal samples from infants with or at risk for NEC [3]–[9]. Many (but not all) of these culture-independent studies have identified differences in the gut microbiota of infants with and without NEC. As seen also with prior culture-based investigations, the findings have varied across studies. Whereas one report identified an abundance of *Clostridium perfringens* within samples from infants with NEC [8], multiple recent studies each identified an abundance of gram-negative facultative anaerobes from the phylum Proteobacteria [4], [6], [7], [10]. Importantly, the identity of the gram-negative organisms observed in the NEC samples has varied significantly within and across studies. Additionally, some studies have noted a reduction in microbial diversity in infants with NEC [7].

An understudied topic has been the composition of mucosa-associated microbial populations within intestinal segments affected with NEC [11], [12]. The goal of this study was to characterize the mucosal-associated microbial populations present within intestinal tissue removed from infants with active NEC. We elected to compare the NEC samples with intestinal tissue removed from hospitalized infants for other indications (referred to as non-NEC samples). As observed in prior studies of fecal samples, we observed high inter-individual variability regarding taxonomic composition within the intestinal tissue of premature infants. We found that NEC samples contained a high total burden of mucosal bacteria and a relative abundance of strict anaerobes, with Proteobacteria being similarly abundant in NEC and non-NEC samples. Analysis of bacterial DNA sequences within these samples indicates that several were colonized by previously uncultured Proteobacteria.

## Methods

### Study subjects

This study was performed with approval from the University of Pittsburgh Institutional Review Board as protocol number PRO09110437 and PRO0606072, and written parental consent was obtained on behalf of the neonates. Per IRB protocol, some samples (e.g. discarded tissue at time of ostomy reversal) were collected without parental consent. All resected intestinal segments were collected from infants hospitalized in the NICU at Children's Hospital of Pittsburgh of UPMC. As per study protocol, the resected tissue was sent for pathologic review and limited portions of the tissue were immediately cryopreserved in sterile tubes for microbial profiling. For this study, the diagnosis of NEC was a pathologic diagnosis made by histologic examination of the resected specimens.

### DNA extraction and 16S rRNA tag sequencing strategy

Microbial DNA was extracted from each sample using the MOBIO (Carlsbad, CA) PowerSoil DNA Isolation kit. Samples were added directly into bead tubes and incubated at 65°C for 10 minutes, and then 60 µL of Solution C1 was added. Tubes were then shaken horizontally for 10 min at maximum speed. All remaining steps followed the manufacturer's protocol. When possible, multiple areas from the resected specimen were included.

We PCR amplified the V2-V4 region of the 16S rRNA gene using primers that incorporated either the A linker, key and a 10 nucleotide barcode or the B linker and key. PCR amplicon libraries were gel purified, quantified, pooled in equimolar ratios, and submitted for pyrosequencing on the Roche/454 GS FLX+ System.

Raw pyrosequencing reads were processed using QIIME software [13], followed by removal of chimeric sequences identified by UChime [14]. QIIME, utilizing Uclust software [15], clustered unique reads at a 0.97 operational taxonomic unit (OTU) threshold, and taxonomic identification was assigned via the Ribosomal Database Project (RDP) classifier [16]. QIIME generated taxa summaries and performed rarefactions, calculated alpha entropy (diversity) within samples, beta entropy between samples, and conducted principal component analysis (PCA).

Comparison of sample composition and identification of statistically significant differences was performed with Microsoft Excel (Redmond, WA). A Bonferroni correction was used when testing multiple hypotheses for taxa differences. Samples were clustered using k-means with silhouette values to visualize stability in Matlab software (MathWorks, Natick, MA), using the first three principle components of beta diversity. The clusters were compared and analyzed in Matlab and Excel.

### Quantitative PCR

To quantify microbial burden within samples, quantitative PCR (qPCR) was performed on DNA extracted from tissue samples from NEC and non-NEC specimens using iQ SYBR Green Supermix (Bio-Rad Laboratories, Hercules, CA) on a CFX96 Touch Real-Time PCR Detection System (Bio-Rad) with universal 16S primers (forward 5′- TAGTCCACGCCGTAAACGATGTCA-3′ and reverse 5′- GCGCACCGTCAATTCCTTTGAGTT-3′). The relative fluorescence units (RFU) and cycle threshold (Ct) were obtained, and then relative Ct values (ΔCt) were calculated relative to a non-NEC sample with minimal 16S Ct value (sample 19). Values were normalized by the mass of tissue used for DNA extraction. Data was then multiplied by a constant (1000), and log-transformed so that it was normal using the Jarque-Bera test.

### Data Deposition

The DNA sequences generated in this study have been deposited in the NIH NCBI SRA database under the accession number SRP040253.

## Results

We analyzed mucosal microbial profiles within 26 intestinal samples (Table 1) resected from 19 infants. Sixteen samples were necrotic bowel segments removed in the setting of fulminant NEC, and these are referred to as NEC cases. Ten samples, referred to as non-NEC cases, were removed for other reasons. In most cases, the non-NEC cases represent tissue samples collected at the time of intestinal anastomosis in infants that previously had required a diverting ostomy due to complicated NEC. Five of the 19 infants had more than 1 sample analyzed.

**Table 1:** Clinical characteristics of infants in this study.

Label	Status	Gestational Age (wks)	Age at surgery (days)	Tissue	Survival	Mode of delivery	Feeding	Perinatal Antibiotics	Preoperative Antibiotic Days	Notes
**1**	NEC	24	15	I	N	CS	C	A, G for 7d	1	
**2**	NEC	36	1	I, C	Y	V	FF	A, G for 1d	1	
**3**	NEC	26	42	I	Y	CS	UNK		10	
**4**	NEC	39	11	I, C, RC	Y	V	BM	A, G for 5d	3	
**5A**	NEC	24	23	I	Y	V	BM	A, G for 10d	3	
**6**	NEC	25	75	I	Y	CS	C	A, G for 7d	1	
**7**	NEC	26	21	I	Y	V	UNK	UNK	18	
**8A**	NEC	29	21	I, C	Y	CS	UNK	UNK	2	
**9A**	NEC	25	12	I, C	N	CS	BM	A, G for 2d	1	
**9B**	NEC	25	14	LC	N	CS	BM	A, G for 2d	3	
**10**	NEC	32	8	RC	Y	V	FF	A, G for 2d	2	
**11A**	NEC	27	17	I	Y	CS	NPO	A, G, Cl for 3d; V, Cf for 14d	17	Peritoneal drain placed for pneumoperitoneum on DOL 4
**12A**	NEC	29	18	I, C	Y	V	UNK	A, G for 2d	2	
**13**	NEC	28	12	I	Y	V	C	A, G for 2d; V, Cf for 2d	3	
**14**	NEC	28	16	J, I, RC	Y	CS	UNK	A, G for 7d	1	
**15**	NEC	33	5	I, RC, TC	Y	V	BM	A, G for 2d	1	
**5B**	non-NEC	24	169	I	Y	V	FF	A, G for 10d	NA	Revision of prior post-NEC ileocolic anastomosis
**8B**	non-NEC	29	44	I	Y	CS	NPO	UNK	15	Delayed creation of ileostomy 23 days after surgery for NEC
**11B**	non-NEC	27	93	I	Y	CS	C	A, G, Cl for 3d; V, Cf for 14d	NA	Ileostomy reversal after NEC
**11C**	non-NEC	27	93	C, RC	Y	CS	C	A, G, Cl for 3d; V, Cf for 14d	NA	Mucus fistula reversal after NEC
**12B**	non-NEC	29	68	I	Y	CS	C	A, G for 2d	NA	Ileostomy reversal after NEC
**12C**	non-NEC	29	68	RC	Y	CS	C	A, G for 2d	NA	Mucus fistula reversal after NEC
**16**	non-NEC	37	1	J	Y	UNK	C	UNK	1	Meconium peritonitis and segmental volvulus
**17**	non-NEC	26	125	I	Y	UNK	UNK	UNK	NA	Ileostomy reversal after NEC
**18**	non-NEC	25	99	J	Y	UNK	BM	A, G for 2d	NA	Jejunostomy reversal after NEC
**19**	non-NEC	25	177	J	Y	UNK	FF	A, G for 7d	NA	Resection of jejunal stricture after medical NEC

Matched samples are indicated by the letters in the label; for example, samples 11A, 11B, and 11C were derived from the same patient. Unknown data is indicated by the abbreviation UNK; NA, not applicable; DOL, day of life.

Abbreviations for tissue types: J, jejunum; I, ileum; C, cecum; RC, right colon; TC, transverse colon; LC, left colon.

Abbreviations for mode of delivery are CS, Cesarean section; V, vaginal delivery.

Feeding refers to enteral nutrition received in the week prior to sample collection, and the abbreviations for feeding are: BM, breast milk; FF, formula feed; C, combination breast and formula; NPO, no feeds.

Perinatal antibiotics refers to the initial course of antibiotics consecutively administered to the baby after delivery. Abbreviations for antibiotics: A, ampicillin; G, gentamycin; V, vancomycin, Cl, clindamycin; Cf, cefotaxime.

Preoperative antibiotic days refer to the number of consecutive days on which antibiotics were administered immediately preceding sample collection. This excludes antibiotics administered at time of surgery.

Mean gestational age of the infants studied was 28 weeks (range 24–39 weeks). NEC cases were obtained from infants with a mean age of 19 days at the time of surgery (range 1–75), whereas non-NEC cases were resected from infants with a mean age of 94 days (range 1–177) (*t*-test, *p* = 0.0016). Two infants did not survive the acute episode of NEC. Three infants underwent surgery within the first week of life; 2 of these had relatively advanced postmenstrual age (33 and 36 weeks) and developed fulminant NEC at 1 and 5 days of life, whereas a third developed a segmental intestinal volvulus with bowel necrosis at 1 day of life. At the time that samples were collected, 4 infants had received broad spectrum antibiotics consecutively for 10 or more days prior to surgery (referred to as high antibiotic, HA), 14 infants had received antibiotics for 4 days or less (low antibiotic, LA), and 8 infants had not been receiving antibiotics (no antibiotics, NA). Most infants also had prior antibiotic exposure during the perinatal period (Table 1). Both the NEC and non-NEC groups contained infants receiving breast milk, infant formula, or a combination of both prior to sample collection (Table 1). Because these patients were transferred to our hospital from a referring institution, medical records regarding perinatal antibiotic exposure and dietary history were incomplete in some instances.

### Overall composition of microbial communities

After processing, a total of 290,590 16S rRNA gene sequences were obtained from the 26 samples on 2 separate 454 pyrosequencing runs. The first sequencing run was conducted at an unusually high depth for quality control purposes, and an average of 26684 sequences per sample (range 18274-36330) was obtained. On the second run, an average of 7484 sequences per sample (range 385-21905) was obtained. The average length per sequence was 534 bp (range 142–743 bp). Rarefaction curves are shown in Figure S1.

**Figure 1:**
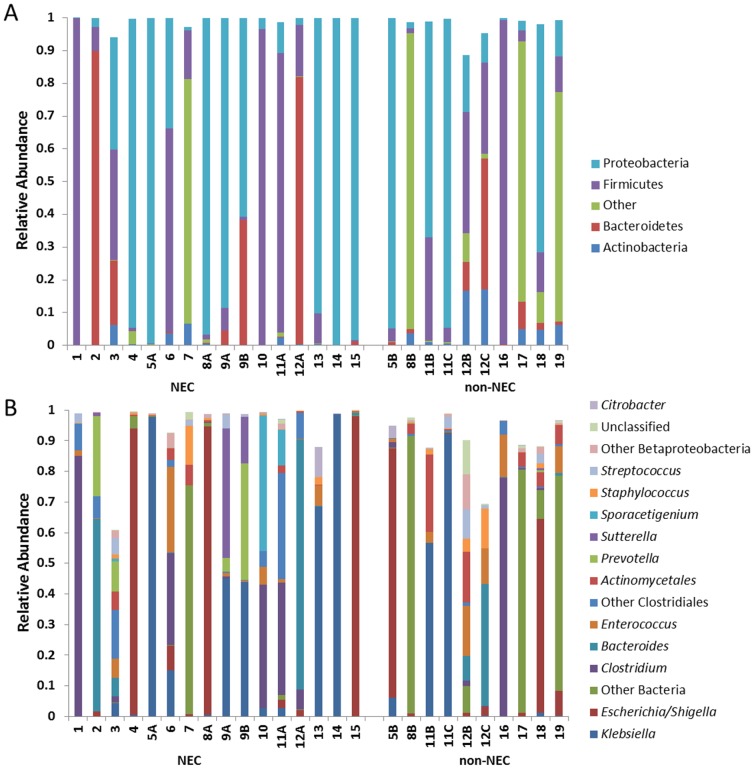
Compositional analysis of mucosa-associated microbial communities in NEC and non-NEC samples. Shown are the relative abundances of bacterial taxa at the (A) phylum and (B) genus levels.

Phylum and genus level taxonomic assignments for all sequences are shown in Figure 1. As shown, 82.7% of sequences were assigned to the three bacterial phyla Proteobacteria (45.5%), Firmicutes (25.7%), and Bacteroidetes (11.5%). An additional 2.9% of sequences were assigned to Actinobacteria, generally from taxa commonly found on the skin (e.g. *Propionibacterium* and *Corynebacterium*). Overall, 11.8% were assigned to the bacterial kingdom but could not be assigned to a phylum. In general, the composition and diversity of mucosa-associated microbial communities observed in this study, specifically the relative abundance of the phyla Firmicutes and Proteobacteria, were similar to fecal microbial communities reported in recent studies of premature infants [3]–[9].

At the genus level, we did not identify any “core” taxa present in all samples. *Escherichia/Shigella*, *Klebsiella*, *Clostridium*, and *Bacteroides* were the most abundant genera overall. Organisms from these genera were present as a major community member (defined as abundance greater than or equal to 1%) in 54%, 50%, 35%, and 31% of all samples, respectively. Remarkably, 24 of the 26 total microbial communities were found to contain at least one dominant member (defined as a taxon representing at least 30% of all sequences) [17], and in several cases the relative abundance of these dominating organisms exceeded 90%. The genera *Escherichia/Shigella* (5 samples), *Klebsiella* (7 samples), *Clostridium* (5 samples), and *Bacteroides* (3 samples) were commonly observed to be dominant. The only other taxa found to be dominant were *Prevotella* (1 sample), *Sporacetigenium* (1 sample) and *Sutterella* (1 sample).

### Comparison of NEC and non-NEC microbial communities

**Figure 2:**
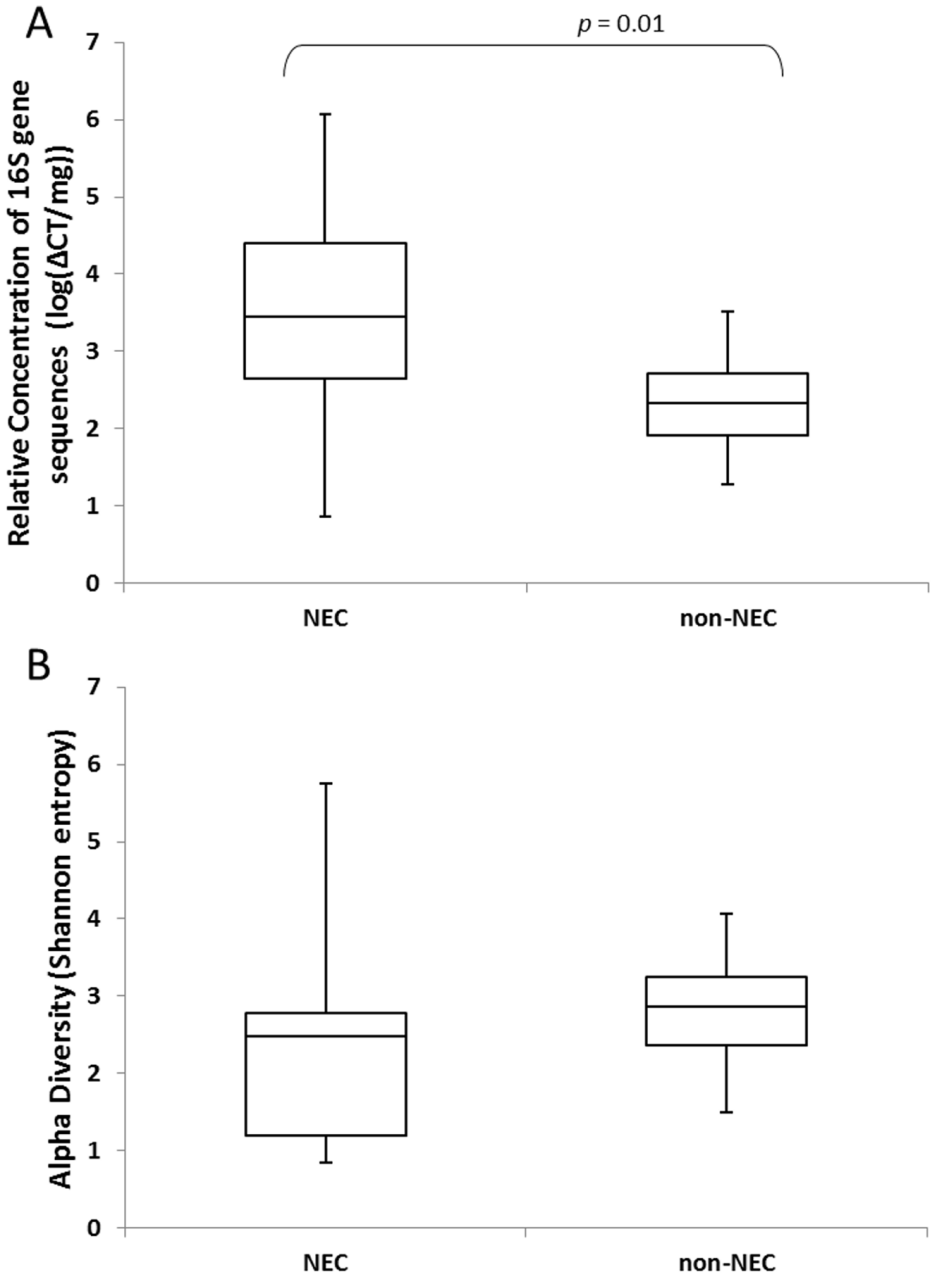
Microbial burden and population diversity. (A) Median relative concentration of 16S gene sequences in NEC and non-NEC samples as determined by qPCR, *p* = 0.01. (B) Median Shannon diversity indices for NEC and non-NEC samples, *p*>0.05. Bars indicate upper and lower quartiles.

**Figure 3:**
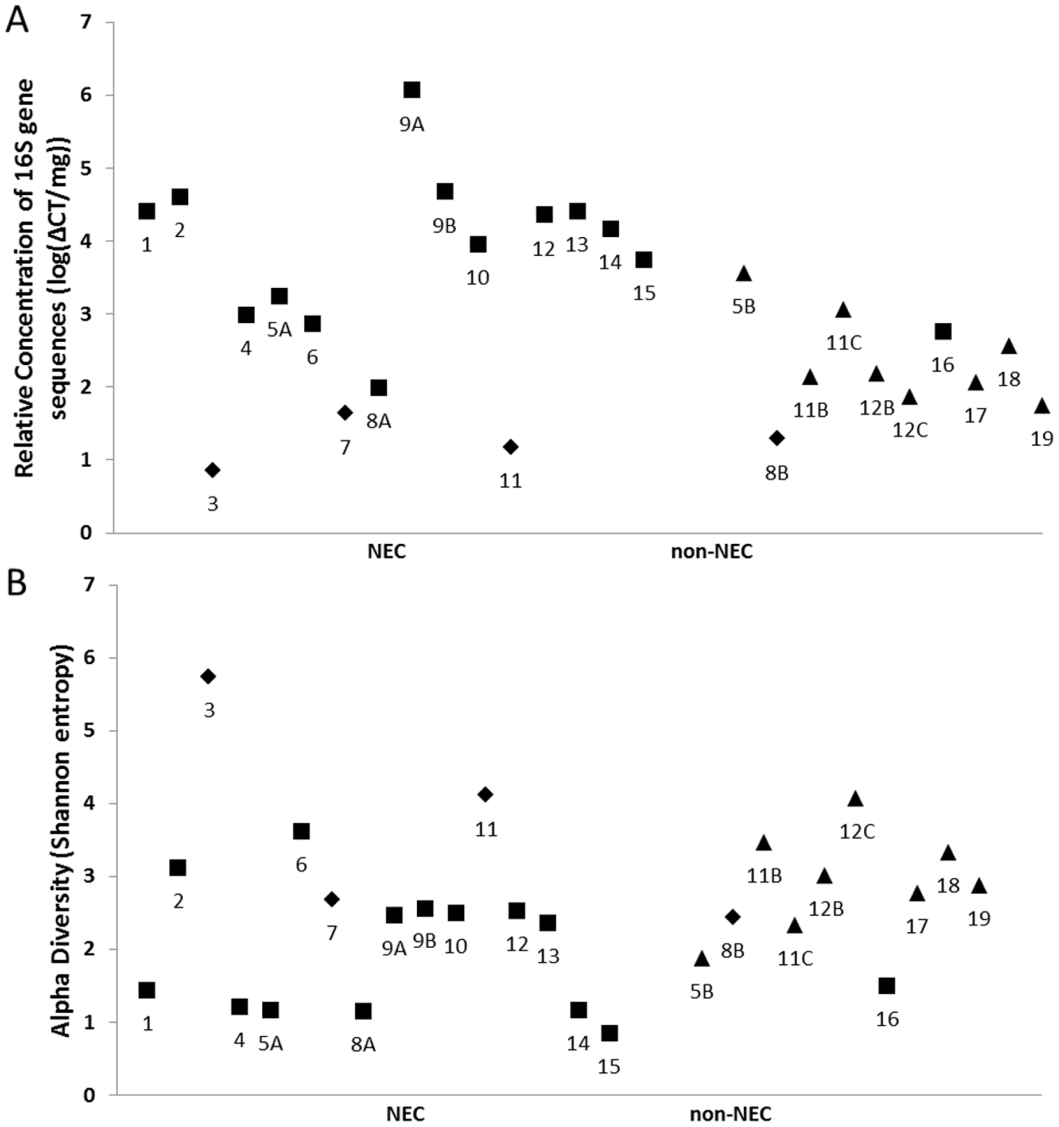
Microbial burden and population diversity. Sample by sample measurements of (A) relative concentration of 16S gene sequences as determined by qPCR and (B) Shannon diversity indices. Shapes indicate heavy antibiotic exposure (diamonds) immediately prior to sample collection, low antibiotic exposure (squares), or no antibiotics (triangles).

Total bacterial load present within the intestinal samples was estimated by measuring the normalized abundance of 16S rRNA gene sequences with qPCR (Figure 2A). The bacterial load of NEC samples was significantly higher than non-NEC samples (*t*-test, *p* = 0.01), despite the finding that the four samples exposed to prolonged preoperative antibiotics (consisting of 3 NEC samples and 1 non-NEC sample) possessed extremely low levels of bacterial DNA (Figure 3A). This supports recent reports that broad-spectrum antibiotic therapy nearly eradicates gut microbial populations [18], and suggests that NEC is associated with local bacterial overgrowth in the absence of an extended exposure to broad-spectrum antibiotics.

Consistent with other reports, we observed high inter-individual variability in the composition of microbial communities in this study. This is illustrated by the fact that several genera (e.g. *Klebsiella*) were dominant in many samples yet absent or nearly absent in other samples. We did not identify any individual taxon (at any phylogenetic level) that was significantly enriched or depleted specifically within NEC samples. *Clostridium* was more commonly observed in the NEC samples than non-NEC, but this relationship did not reach statistical significance. Interestingly, only 1 non-NEC sample contained clostridial species at a significant level (sample 16). This sample was removed from an infant with segmental intestinal volvulus with bowel necrosis, indicating that the presence of clostridial species in intestinal resection may be a nonspecific finding associated with ischemia and necrosis rather than NEC.

Microbial diversity also varied significantly across infants. No significant difference was observed in the mean Shannon diversity index (SDI) for the NEC and non-NEC groups (*t*-test, *p*>0.05) (Figure 2B). Interestingly, we observed that the high antibiotics group tended to have very high SDI despite the exceedingly low total bacterial burden (Figure 3B). When the 4 HA samples were removed from the analysis, the mean SDI was significantly lower in the NEC group than the non-NEC group (*t*-test, *p* = 0.04).

### Detection of specific clusters of samples

**Figure 4:**
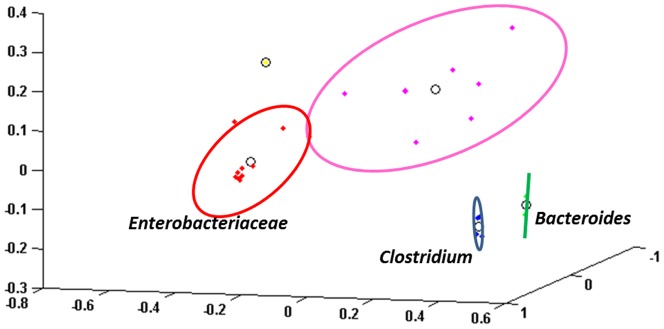
K-means clustering according to beta diversity. K-means clustering of NEC and non-NEC samples, according to beta diversity. Individual data points represent individual samples. The ovals around the points represent 2 standard deviations of the data, and the circle in the middle of each oval represents the center of the cluster. The *Enterobacteriaceae* cluster is shown in red, the *Clostridium* cluster in blue, and *Bacteroides* cluster in green. The single yellow point represents sample 9B, and the magenta points form a heterogenous cluster.

To further assess the similarities and differences in community composition across samples, we used principal component analysis and k-means clustering of the first 3 principal components of the beta diversity (k = 5) [19] (Figure 4). These clusters are generally distinguished by the variation in the abundance of one of three bacterial families: *Enterobacteriaceae* (11 samples within the cluster), *Clostridiaceae* (4 samples), and *Bacteroidaceae* (2 samples). A fourth cluster contained only a single sample (sample 9B), dominated by both *Prevotella* and *Klebsiella*. A final large cluster was the most heterogeneous and least defined, containing 8 samples marked by an abundance of bacterial sequences that could not be confidently identified even at the phylum level.

To further define these clusters, we attempted to annotate individual taxa of interest with blastn searches against the curated Bacteria database of RDP. The *Clostridiaceae* cluster contained 4 NEC samples and the intestinal volvulus sample. Two of the NEC samples from this cluster contained *C. perfringens*, which has frequently been observed in association with NEC [8], [20], 1 sample contained *C. paraputrificum*, and one sample contained both. *C. perfringens* was also observed at high relative abundance (30%) in a NEC sample assigned to cluster 5, and was observed at low abundance in NEC samples 3 and 12, and non-NEC sample 5B. Interestingly, there were two distinct *C. perfringens* OTUs present within samples 11A, 12A and 5B, suggesting the presence of distinct and co-existing strains.

The *Bacteroidaceae* cluster contained 2 NEC samples marked by a high abundance of *Bacteroides*. From these 2 samples, we observed 3 distinct *Bacteroides* OTUs. Each was labeled as “uncultured” and species-level assignments could not be made. All samples dominated by obligate anaerobes (*Clostridium*, *Bacteroides*, *Prevotella*, and/or *Sporacetigenium*) were NEC samples with the exception of the intestinal volvulus case.

The *Enterobacteriaceae* cluster was the largest. Interestingly, the mean abundance of the most dominant organism was greater than 90% for 5 of the NEC samples, but for only 1 of the non-NEC samples in this cluster. The SDI for the NEC samples within this cluster was significantly lower than the non-NEC samples (*t*-test, *p* = 0.035). Six samples were dominated by OTUs labeled by RDP as “uncultured” *Klebsiella* species (mean abundance 76.49%, range 44.99–98.6%) and 5 were dominated by OTUs labeled by RDP as “uncultured” *Escherichia/Shigella* species (mean abundance 86.07%, range 63.21–98.02%). Both NEC samples from infant 9 were marked by the presence of a *Sutterella* organism (phylum Proteobacteria) that also could not be successfully identified at the species level. These results indicate that both NEC and non-NEC samples harbor a high abundance of poorly characterized members of otherwise common gram-negative bacterial genera.

### Intra-individual variation

**Figure 5:**
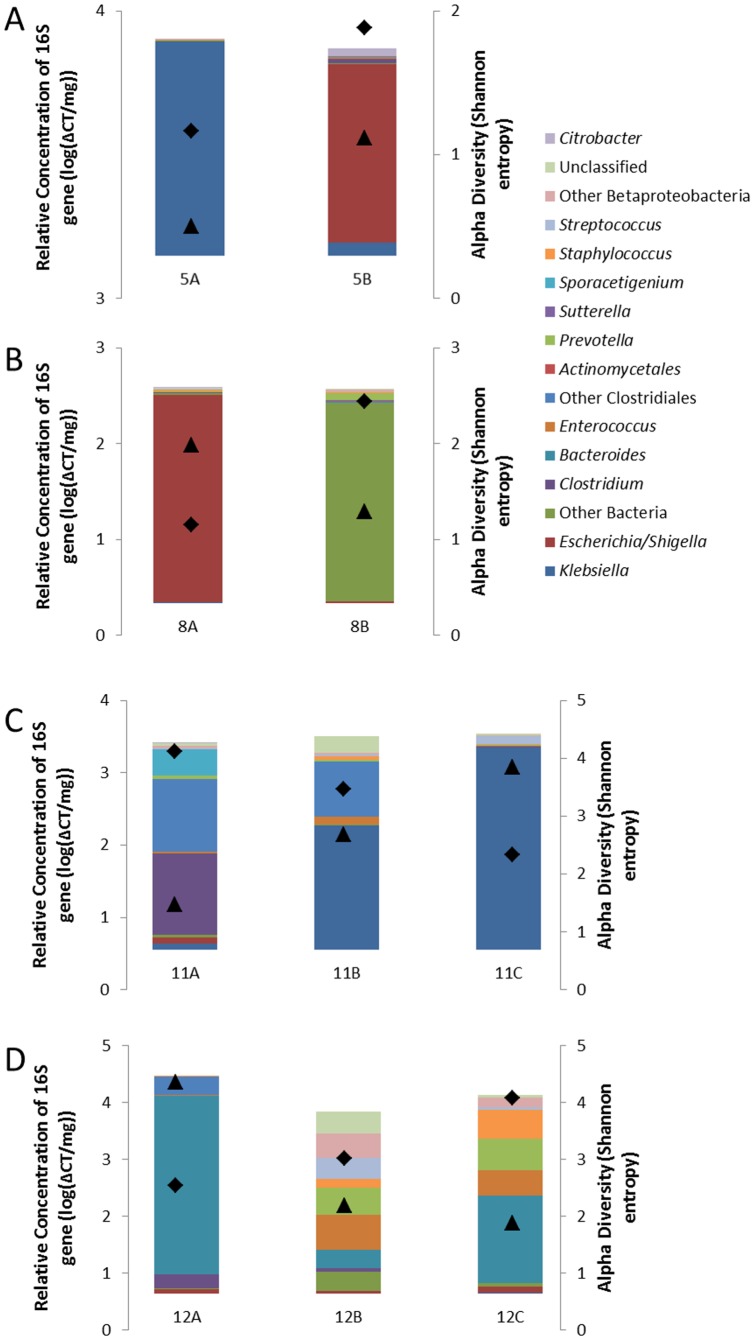
Temporal and spatial patterns of intraindividual variation. Represented here are all samples from the four infants (panels A-D) with both NEC and non-NEC samples. Colored bars represent genus-level taxonomic assignments corresponding to bars shown in Figure 2. Superimposed symbols represent relative concentration of 16S rRNA genes (triangles) and Shannon diversity index (diamonds).

Five infants had more than 1 sample collected, and analysis of these samples allowed for exploration of temporal and spatial patterns of intraindividual variation (Figure 5). Infant 5 (Figure 5A) had 2 samples of the ileum; the first sample was necrotic and sampled at diagnosis of NEC and a second sample of grossly normal ileum was collected at the time of ileostomy reversal. Both samples were within the *Enterobacteriaceae* cluster, but the first was dominated by *Klebsiella* and the second was dominated by *Escherichia*/*Shigella*. Additionally, the alpha diversity increased from 1.16 to 1.88. An initial sample from infant 8 (Figure 5B) was an ileocecectomy NEC specimen in the *Enterobacteriaceae* cluster, but the subsequent ileum specimen was markedly distinct with a high abundance of bacterial sequences that could not be assigned at the phylum level. The alpha diversity present increased from 1.15 initially to 2.44.

From infant 11 (Figure 5C), an ileal sample enriched in anaerobic genera *Clostridium* and *Blautia* (anaerobe from the class Clostridiales) was obtained at the time of the initial surgery. At the time of ileostomy reversal, non-NEC samples from the ileostomy and the defunctionalized mucous fistula were found to be very similar to each other (both within the Proteobacteria cluster, enriched for *Klebsiella*) but were significantly different than the NEC sample. A similar transition was seen for infant 12 (Figure 5D). The NEC sample was enriched for *Bacteroides*; the non-NEC samples collected during anastomosis of the ileostomy and mucous fistula harbored a large population of organisms that could not be classified and only a small population of *Bacteroides* (81.6% vs. 8.19%). Interestingly, the NEC sample for infant 11 was in the high antibiotic group with high alpha diversity and a low relative 16S rRNA concentration relative to the subsequent non-NEC samples. By contrast, infant 12 had increased alpha diversity after recovery from NEC while the relative 16S rRNA concentration decreased.

Finally, infant 9 underwent 2 separate surgeries over the period of 72 hours for active NEC. This infant did not survive, and therefore no subsequent samples were collected. A first ileocectomy sample contained 2 dominant taxa from the phylum Proteobacteria (*Klebsiella* and *Sutterella*) in addition to the genera *Streptococcus* (4.4% relative abundance) and the strict anaerobe *Prevotella* (4.4%). Two days later, a necrotic segment of the left colon was removed. This second sample also contained a large amount of *Klebsiella*, but the relative abundance of *Sutterella* decreased to 15.2% and the relative abundance of *Prevotella* increased dramatically to 38.0%. It is not possible to determine whether these differences reflect spatial diversity along the gastrointestinal tract, or the sequellae of ongoing systemic disease or antibiotic therapy. Of note, *Prevotella* was observed in samples from only 2 other infants, and both of these were NEC cases.

## Discussion

Several lines of evidence support the hypothesis that bacteria contribute to NEC pathogenesis [2]. These include: endotoxemia in affected infants, efficacy of antibiotics in a majority of cases, presence of *pneumatosis intestinalis*, and the occurrence of well-documented disease outbreaks in which multiple infants within a single ICU concomitantly develop the disease. For nearly 4 decades, investigators have published the results of efforts to identify specific organisms that “cause” NEC. The findings of our study suggest that there is not a single causative organism associated with NEC or even a single pattern of microbial diversity associated with the disease. A key finding of recent studies, and one that sharply impacts statistical analyses, has been the extraordinary intraindividual and interindividual variability regarding microbial colonization patterns in infant samples. This variability far exceeds that seen in adults and older children [21], and as a result it is common to observe organisms that are highly abundant in several samples yet completely absent from others. This variability must be considered in interpreting observational studies of infant microbiota.

The current study utilized resected segments of intestinal tissue from newborn infants that were prospectively collected and cryopreserved specifically to examine microbial diversity within the samples. The results of our study line up closely with those by Smith *et al*. [12], in which formalin-fixed, paraffin-embedded NEC tissues were analyzed retrospectively to characterize NEC-associated microbial populations. Both studies agree with another report [22] that NEC tissue samples generally contain a high burden of bacteria with the exception of tissues collected from infants that had been recently exposed to prolonged broad-spectrum antibiotics. Additionally, both studies showed a higher relative abundance of Proteobacteria and Firmicutes than other bacterial phyla. This is consistent with patterns of diversity seen in fecal samples from newborn premature infants, but differs from fecal samples from older children and adults that are enriched with both Bacteroidetes and Firmicutes but typically harbor smaller numbers of Proteobacteria [23].

It has been proposed that NEC represents a final common inflammatory pathway resulting from more than 1 pathologic process [20]. The current study provides evidence that NEC samples display 1 of at least 2 distinct patterns: *either* the samples contain high amounts of obligate anaerobes, *or* they contain high amounts of Proteobacteria. This observation appears to be independent of the location within the GI tract from which samples are collected. In the case of anaerobes, it is intriguing that almost none of the non-NEC samples contained significant populations of strict anaerobes despite the fact that some samples were harvested from infants at several months of age. The only exception was a non-NEC sample removed from the 1-day-old term infant with intestinal volvulus and necrosis; over 82% of sequences from this sample belonged to the class Clostridiales. By contrast, we found several NEC samples harboring dominant proportions of *Clostridia*, *Bacteroides*, or *Prevotella* and/or significant proportions of the anaerobes *Blautia* and *Sporacetigenium*. This suggests that strict anaerobes are not normally present within the intestinal wall of newborn infants in the absence of a severe ischemic or inflammatory process. In the Smith study, it was observed that *pneumatosis intestinalis* was only present in cases dominated by Clostridia, but our study did not reproduce this finding (data not shown). The absence of strict anaerobes in the non-NEC cases could be partially explained by the presence of ostomies in many of these cases, since it has been suggested that ostomies convert the gut ecosystem from an anaerobic to an aerobic system [24]. Historically, recommended antibiotic regimens for NEC have not always included agents with strong anaerobic coverage. We do not advocate any changes in clinical practice or antibiotic selection based upon this study, but these data point to the importance of further research to optimize antibiotic coverage in infants with suspected or documented NEC.

Whereas anaerobes were predominantly observed only in diseased tissue, a high abundance of Proteobacteria was observed in both non-NEC tissue samples and NEC samples. While some recent studies have agreed with this finding, several others have reported a statistically significant increase in the abundance of Proteobacteria in fecal samples from babies with NEC. In our study, a notable finding was that NEC samples dominated by Proteobacteria had significantly lower community diversity than the non-NEC samples, a finding also reported by some [7] but not others [6]. In two infants with NEC (subjects 5 and 8), in whom NEC samples were dominated by Proteobacteria, temporal monitoring demonstrated that recovery from NEC was associated with a subsequent increase in community diversity.

In parallel with observational studies of infant microbiota such as this, recent mechanistic studies have begun to clarify how disordering of the host-microbe relationship may be associated with intestinal disease. With regard to Proteobacteria, publications have clearly documented how expansion these populations of gram-negative organisms can either cause gut inflammation [25] or alternatively result secondarily from gut inflammation [26]. Taken together, these considerations help illustrate the complexity of associations between NEC and the abundance of Proteobacteria, and also illustrate the need to further monitor temporal patterns of colonization.

The potential link between NEC and obligate anaerobes may be completely distinct from the above considerations regarding Proteobacteria. The Clostridiales are a diverse and poorly understood group of bacteria. It is clear that clostridial species are common in fecal samples from premature infants near the time of discharge, but they are seemingly absent during the first weeks of life [27]. Aside from the current study and the Smith study, little is known about the clostridial burden within intestinal tissue from premature infants. However, the potential for some clostridial species, particularly *C. perfringens*, to cause fatal intestinal inflammation in animal models and some clinical settings is well established, and so we agree that further work is required to address the longstanding hypothesis [28] that increased abundance of *C. perfringens* colonization is associated with risk of NEC. At the same time, it must be acknowledged that some commensal clostridial species have been shown in animal models to be essential for development of the immune system in the newborn period and for protection from infection [29]. Other anaerobes, e.g. *Bacteroides* species, have not been commonly observed in the premature infant gut during the first weeks of life [5], [30]. Thus it was somewhat surprising to observe the presence of a poorly characterized *Bacteroides* species at high abundance in NEC tissue only. This suggests that an abundance of strict anaerobes very early in the life of a premature infant may be a risk factor for NEC. In two infants with NEC samples dominated by strict anaerobes, it was interesting to note that their recovery from NEC involved a transition to microbial communities with fewer obligate anaerobes.

An ideal control group for this study would be a cohort of age-matched premature infants undergoing intestinal resections for diagnoses other than NEC, e.g. atresia or Hirschsprung's disease. However, such infants are exceedingly rare. Another nice control group would be matched preoperative fecal samples from infants undergoing surgery. These samples are also difficult to collect, particularly since many babies requiring surgery for NEC at our institution are transferred from referring facilities. For these reasons, rather than including a formal “control” group, we elected to include a set of “non-NEC” samples from a range of hospitalized newborn infants that could serve as a basis for comparison with NEC samples. We acknowledge the limitations inherent with this comparison. Two important confounding variables impacting this comparison are age and antibiotic exposure at the time of surgery. It has clearly been demonstrated that each of these variables can impact the structure of gut microbial communities [31], [32]. Additionally, infants with NEC generally do not receive enteral nutrition in the days prior to surgery, and this likely represents a relevant consideration. As with other complex problems in critically ill patients, it will be challenging to tease apart these clinical variables without a significantly larger group of study subjects that might allow for a regression analysis of relevant covariates.

By documenting which organisms are present within intestinal segments resected from newborn infants, this study adds to knowledge about gut bacteria in infants with and without NEC. Overall, it supports the increasingly accepted idea that taxonomic identifications of organisms present within clinical samples are probably inadequate to fully resolve this relationship [33]. This task will likely require higher resolution studies of bacterial function. Given the recent advent of next-generation high-throughput platforms, it may now be possible to develop these approaches and hopefully to improve clinical outcomes for newborns at risk for NEC.

## Supporting Information

Figure S1 Mucosa-associated bacterial diversity. Rarefaction analysis demonstrating increasing number of phylotypes observed with increasing depth of sequencing.
(DOCX)
