# Supplementary material for: Mucosa-Associated Bacterial Diversity in Necrotizing Enterocolitis
Source: PLoS One. 2014 Sep 9;9(9):e105046. doi: 10.1371/journal.pone.0105046 (PMC4159227; doi:10.1371/journal.pone.0105046)

**Supplemental Figure 1. Mucosa-associated bacterial diversity.** Rarefaction analysis demonstrating increasing number of phylotypes observed with increasing depth of sequencing.


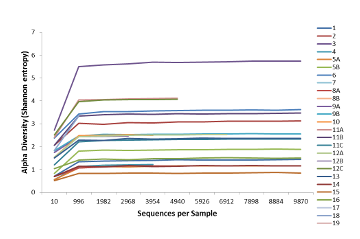

Supplement: Figure S1 — Mucosa-associated bacterial diversity. Rarefaction analysis demonstrating increasing number of phylotypes observed with increasing depth of sequencing. (DOCX) [file pone.0105046.s001.docx]
